# Mifepristone and rapamycin have non-additive benefits for life span in mated female *Drosophila*

**DOI:** 10.1080/19336934.2024.2419151

**Published:** 2024-10-23

**Authors:** Gary N. Landis, Britta Baybutt, Shoham Das, Yijie Fan, Kate Olsen, Karissa Yan, John Tower

**Affiliations:** Molecular and Computational Biology Section, Department of Biological Sciences, University of Southern California, Los Angeles, CA, USA

**Keywords:** Drosophila, ageing, mifepristone, rapamycin, steroid, mitophagy, plasticity, hypertrophy, midgut, sex peptide

## Abstract

The drugs mifepristone and rapamycin were compared for their relative ability to increase the life span of mated female *Drosophila melanogaster*. Titration of rapamycin indicated an optimal concentration of approximately 50 μM, which increased median life span here by average +81%. Meta-analysis of previous mifepristone titrations indicated an optimal concentration of approximately 466 μM, which increased median life span here by average +114%. Combining mifepristone with various concentrations of rapamycin did not produce further increases in life span, and instead reduced life span relative to either drug alone. Assay of maximum midgut diameter indicated that rapamycin was equally efficacious as mifepristone in reducing mating-induced midgut hypertrophy. The mito-QC mitophagy reporter is a previously described green fluorescent protein (GFP)–mCherry fusion protein targeted to the outer mitochondrial membrane. Inhibition of GFP fluorescence by the acidic environment of the autophagolysosome yields an increased red/green fluorescence ratio indicative of increased mitophagy. Creation of a multi-copy mito-QC reporter strain facilitated assay in live adult flies, as well as in dissected midgut tissue. Mifepristone was equally efficacious as rapamycin in activating the mito-QC mitophagy reporter in the adult female fat-body and midgut. The data suggest that mifepristone and rapamycin act through a common pathway to increase mated female *Drosophila* life span, and implicate increased mitophagy and decreased midgut hypertrophy in that pathway.

## Introduction

Mifepristone (RU486) is a synthetic steroid drug with a long record of safe and effective use in humans. Mifepristone antagonizes the type II glucocorticoid receptor (GR), enabling its use as a treatment for Cushing’s syndrome (hypercortisolism) [1]. Mifepristone also antagonizes the progesterone receptor, enabling its use for birth control [2]. In addition, mifepristone is an agonist of mammalian peroxisome proliferator-activated receptor gamma (PPARγ), which is a ligand-inducible transcription factor that functions in a variety of cell types to regulate metabolism, immune function and cell proliferation [3–5]. Mifepristone has been found to have anti-obesity and anti-diabetic effects in human and rodent models, including reducing serum triglyceride levels, and both GR and PPARγ are implicated as relevant targets for these beneficial metabolic effects [6–12].

Mifepristone also shows promise as a treatment for alcohol abuse disorder, endometriosis, cancer and other conditions [2,13–15]. Alcohol abuse disorder is a complex psychiatric condition involving changes in brain reward and stress systems [16]. Studies in rodent models implicate dysregulation of the hypothalamic–pituitary–adrenal axis associated with altered GR expression [14]. Mifepristone treatment is reported to reduce alcohol consumption in rodent models and in human subjects [14,17], consistent with a mechanism involving antagonism of the GR receptor. Endometriosis is caused by proliferation of endometrial tissue outside the uterine cavity [18]. By antagonizing the PR, mifepristone inhibits proliferation of endometrial tissue, thereby providing a partly effective intervention. The anti-cancer effects of mifepristone have been found to be multi-faceted. Mifepristone antagonism of the GR is implicated in its ability to inhibit growth of certain cancer cells and tumours bearing an active form of this receptor, including metastatic melanoma [19]. Similarly, mifepristone antagonism of the PR is implicated in its ability to inhibit growth of certain cancer cells and tumours bearing an active form of this receptor, including metastatic breast cancer [20,21]. However, mifepristone also inhibits the growth of a variety of PR-negative cancer cells and tumours through mechanisms that remain unclear [22]. In summary, the clinical efficacy of mifepristone involves its known targets, as well as targets that remain to be determined. Given the steadily increasing use of mifepristone in humans, it will be important to understand its potential effects on ageing.

Mifepristone also has a variety of physiological effects in *Drosophila*. In response to mating, female *Drosophila* undergo a change in physiology, including midgut hypertrophy [23–25]. These changes are thought to support increased egg production, but also come at a cost of decreased life span. Feeding the mated females with mifepristone has been found to block these effects of mating. In mated female *Drosophila*, mifepristone dramatically increases life span, and reduces midgut size, triglyceride levels, amino acid (AA) metabolites, egg production and inflammation [24,26,27]. In virgin female *Drosophila*, mifepristone yields smaller increases in life span, and also reduces triglyceride levels, AA metabolites and egg production, but does not detectably affect midgut size [28,29]. Several different methods have been used to assay the effect of mifepristone treatment on food intake in mated female *Drosophila* and virgin female *Drosophila*, including the dye-uptake assay [24], the capillary feeding assay (CAFE) [26] and the excrement quantification (EXQ) assay [27,28,30], and in each case, there was no decrease in food intake, and often a significant increase in food intake. These results indicate that mifepristone life span extension is not due to reduced food intake. In male *Drosophila*, mifepristone reduces triglyceride levels, but does not increase life span [24,29].

Mitophagy refers to a specific form of macroautophagy that degrades damaged and superfluous mitochondria [31]. The mitochondria become engulfed in a double-membrane vesicle called the autophagosome, which then fuses with the acidic lysosome to create the autophagolysosome. In the acidic environment of the autophagolysosome, the mitochondria are degraded by lysosomal hydrolases. Increasing evidence suggests that impaired mitophagy may play a causative role in human ageing and ageing-associated disease, including neurodegenerative disease and gastrointestinal disease [32,33]. Engineered fluorescent reporter proteins, including mitochondrial quality-control (mito-QC) have been particularly useful in the study of mitophagy [34,35]. Mito-QC is a fusion of the GFP and mCherry fluorescent proteins, targeted to the outer mitochondrial membrane by a fusion to the transmembrane domain from mitochondrial fission protein 1. Mito-QC marks intact mitochondria with both green and red fluorescence. Exposure to the acidic environment of the autophagolysosome quenches the green fluorescence, and therefore an increase in red/green fluorescence ratio reveals increased mitophagy [35]. Activation of mitophagy by small molecules, including rapamycin, has been reported to inhibit cellular senescence and shows promise as an intervention in ageing-associated disease [36–38].

Rapamycin (sirolimus) is a macrocyclic lactone first isolated from *Streptomyces hygroscopicus* [39]. In eukaryotic cells, rapamycin binds the factor FKBP12, and this complex in turn inhibits the conserved mTOR (mechanistic target of rapamycin) pathway [40–42]. The mTOR pathway integrates multiple inputs, including nutrient availability and growth factor signalling. In response, the mTOR pathway positively regulates protein synthesis and cell growth, and negatively regulates all forms of macroautophagy, including mitophagy [43]. Rapamycin has been shown to increase life span in mice, with relatively greater efficacy in females relative to males [44,45]. Rapamycin also shows favourable effects on ageing phenotypes in humans [46,47].

In *Drosophila*, rapamycin has been reported to extend the median life span of adult mated female flies by approximately + 25% [48]. Rapamycin was initially reported to produce a small but significant increase in male life span [48], but a subsequent analysis found no significant effect in males [49]. Both *Drosophila* midgut tissue and fat-body tissue have been implicated as critical targets for the beneficial effects of rapamycin on life span in mated females. Inhibition of autophagy by tissue-general or enterocyte-targeted expression of ATG5-RNAi eliminated the life span benefits of rapamycin, suggesting that upregulation of autophagy in midgut is required for life span extension by rapamycin [48]. However, the potential role of mitophagy has not been specifically addressed. Rapamycin is reported to decrease midgut intestinal stem cell (ISC) proliferation in mated female flies [50], however, whether this results in altered midgut size remains unclear. The mTOR pathway mediates increased protein synthesis by activating the translation regulator ribosomal protein S6 kinase (S6K). Inhibition of S6K activity , specifically, in fat-body tissue was reported to extend mated female fly life span [51], and to eliminate the benefit of rapamycin for mated female life span [52].

The physiological effects of mifepristone and rapamycin in *Drosophila* have several common features. Both drugs increase life span in females but not males, both drugs are associated with alterations in midgut physiology, both drugs reduce egg production [24,29,48], and both drugs increase life span independent of the microbiome [53,54]. These similarities suggested the possibility that the two drugs might increase life span through the same pathway. To begin to address this hypothesis, experiments were conducted to compare the life span effects of the drugs alone and in combination.

## Materials and methods

### Drosophila strains, culture, drug treatments and life span assay

*Drosophila melanogaster* flies were cultured and life span assays conducted in Percival brand incubators, at 25°C, 12:12 h light-dark cycle, at 65–80% humidity, using a standard agar/dextrose/corn meal/yeast media [55]. Several *Drosophila* strains were obtained from the Bloomington *Drosophila* Stock Center, including: strain y[1] w[*]; P{w[+mC]=elav-Switch.O}GSG301 (BDSC#43642), abbreviated here as *yw;ElavGS*, strain y[1] w[*]; P{w[+mC]=tubP-GAL4}LL7/TM3, Sb[1] Ser[1] (BDSC#5138), abbreviated here as *yw;Tub-GAL4*, strain w[*]; P{w[+mC]=ppl-GAL4.P}2 (BDSC#58768), abbreviated here as *w;ppl-GAL4*. The *w[1118]* strain is the isogenized version (*w[1118]-iso; 2-iso; 3-iso*). The *w[1118]* strain was previously cured of *Wolbachia* by three generations treatment with doxycycline, with confirmation using polymerase chain reaction and *Wolbachia*-specific primers [56]. To generate flies for life span assays, *w[1118]* strain males were crossed to *yw;ElavGS* strain virgins, and the female progeny were collected as virgins over 24 h. These flies were either assayed as virgins, or were mated for 48 h to young (5–14 days of age) *w[1118]* strain males at a ratio of 20 males to 20 females. After mating the males were removed, and the virgin female and mated female flies were maintained in culture vials with 20 flies per vial, in the presence/absence of drug, as indicated. Drugs were administered as previously described [55,57], by adding 50 μl of 20X stock solution in ethanol, evenly to the surface of the vial, and allowing it to absorb and dry overnight. Final concentration of drug in the media was calculated based on absorption into the top ~1 ml of media, as determined by dye-absorption controls [55,57]; control vials received equal volume of ethanol vehicle, and all vials were allowed to dry overnight. Mifepristone (RU486) was obtained from Sigma-Aldrich (cat. #M8046), and flies were treated with 200 μg/ml (466 μM) final concentration in the media. All flies were transferred to fresh vials every other day, and the number of dead flies was recorded. Median life span, percent change in median, and log-rank tests were conducted using R statistical environment [58]. Log-rank analysis was corrected for multiple comparisons using Bonferroni correction, and the *p* value for significance at 5% error rate is indicated in the figure legends. Cox regression with Efron’s approximation was conducted using Prism version 10.3.1 for Mac, GraphPad Software, Boston, Massachusetts, USA, www.graphpad.com.

### Egg laying assay

Mated female flies were generated as described above, and egg production was quantified every other day from day 2 of drug treatment to day 20 of drug treatment. For each group, 5 vials of 20 flies each were assayed at each time point, and the dissecting microscope was used to count total eggs laid per vial over 24 h, with egg laying beginning at ~11 AM and ending at the same time on the next day. Area under curve (AUC) analysis was used to estimate total eggs laid over the entire assay period for each of the 5 replicate vials. The average and standard deviation of the AUC values is presented in bar graphs, normalized to the control (no drug) group. Unpaired, two-tailed t tests were used to determine any significant differences in AUC between samples using Prism 10. A Bonferroni correction was used to control for multiple comparisons, and the *p* value for significance at 5% error rate is indicated in the figure legends.

### Generation of a multi-copy UAS-mito-QC strain

The *UAS-mito-QC* strains, with insertions on the second and third chromosomes, respectively, were generously provided by Alexander Whitworth, and have been previously described [35]. The second chromosome insertion strain was crossed to the delta2-3 transposase strain [59] to mobilize the insertion (Supplemental Figure S1). A duplication of the insertion on the second chromosome was identified by increased expression of the mini-*white*+ marker gene, confirmed by doubled expression of red and green fluorescence when crossed to *yw;Tub-GAL4* driver strain, and named y*w*; UAS-mitoQC2-2. The same strategy was used to double the copy number of the third chromosome insertion, to generate a strain named y*w*; UAS-mitoQC3-2. The second and third chromosomes bearing the multi-copy insertions were then combined using a double-balancer to generate the final strain containing eight copies of the construct, named y*w*; UAS-mitoQC2-2; UAS-mitoQC3-2.

### Maximum midgut diameter assay

Virgin and mated female flies were generated as described above, the males were removed, and then the females were maintained in culture vials in the presence or absence of drug for 12 days. Maximum midgut diameter was assayed as previously described [28]. Briefly, midguts were dissected in phosphate-buffered saline solution in groups of 5 flies at a time, and immediately mounted on slides with coverslip spaced using double-stick tape [60]. Visible light images were generated and analysed using ImageJ [61]. The maximum diameter region of each midgut sample was estimated by inspection, multiple measurements in that region were generated using ImageJ, and the largest value was used for analysis. Unpaired, two-tailed t tests were conducted using Prism 10, and any outliers were identified using Grubbs test. Multiple comparisons were controlled using Bonferroni correction, and the *p* value for significance at 5% error rate is indicated in the figure legends.

### Fluorescence image capture and ImageJ quantification

Fluorescence images were generated using the Leica MZFLIII fluorescence stereomicroscope and Spot imaging software [62]. To generate images of midgut and hindgut, the tissue was first isolated and mounted on slides, as described above. To generate images of live flies, the flies were anesthetized with humidified CO_2_ gas, and photographed on the flypad. Images were captured in the green fluorescence channel and the red fluorescence channel for each sample. For ImageJ quantification, the green channel and red channel images were opened at the same time for each sample. The free-hand drawing tool was used to outline the region of interest (ROI) on the green channel image (which was typically the brighter of the two images), and mean pixel intensity was quantified. The ROI manager was then used to transfer the exact same ROI outline to the red channel image, and mean pixel intensity was quantified. For assay of fluorescence in whole flies, the eyes, wings and any regions of the legs that extended beyond the boundary of the body outline were excluded from the ROI. The data is plotted as the ratio of the red/green mean pixel intensity for each sample. Groups were compared using unpaired, two-tailed t tests conducted with Prism 10, and any outliers were identified using Grubbs test. Multiple comparisons were controlled using Bonferroni correction, and the *p* value for significance at 5% error rate is indicated in the figure legends.

## Results

### Mifepristone and rapamycin have non-additive benefits for median life span

Rapamycin was tested at various concentrations for the ability to increase life span of mated female *Drosophila*, both alone and in combination with mifepristone. The flies used for the life span assays are the hybrid progeny of a cross of two common laboratory strains, the *w[1118]* strain and the *yw;ElavGS* strain. The hybrid progeny from this cross have a longer starting life span than do the homozygous progeny from either parent strain, and previous studies suggest that a larger life span effect of mating and of mifepristone is observed in genotypes with a longer starting life span [24]. The *w[1118]* strain and the *yw;ElavGS* strain were chosen for this purpose because both strains are healthy and easy to maintain, and multiple previous studies show that the female progeny from this cross have an especially long starting life span, and exhibit a large negative change in life span in response to mating and a large positive change in life span in response to mifepristone [24,26,28].

**Figure 1.**
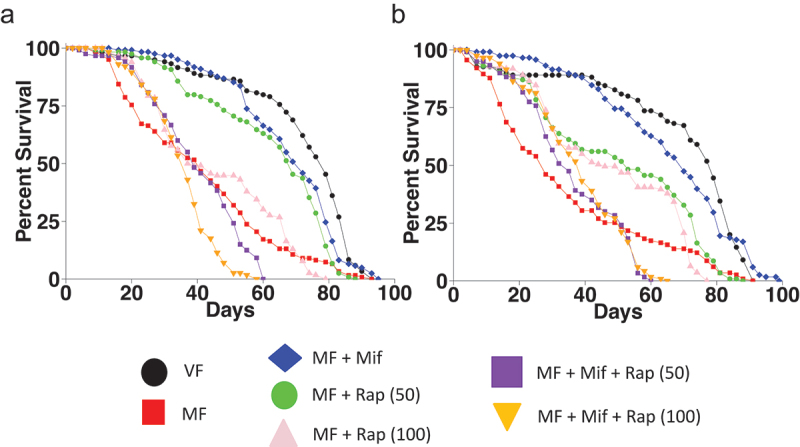
Mifepristone and rapamycin have non-additive benefits for mated female life span. Assay of rapamycin at 50 μM and 100 μM. Life span was assayed in virgin females (VF), mated females (MF), and in mated females treated with mifepristone (mif, 466 μM), and/or rapamycin (Rap), with the μM concentrations of rapamycin indicated in parentheses. (a) Experiment 1. (b) Experiment 2. Statistical summary is presented in Table 1, and Cox-PHA is presented in Table 2.

First, mated females were compared to virgin females to show the decrease in median life span caused by mating, in two replicate experiments (Figure 1; Table 1). Relative to virgin females, mating yielded decreases of −48% and −65%, respectively. Or alternatively, expressed relative to mated females, abstention from mating (virginity) yielded increases of 92.7% and 182%, respectively. Treatment of the mated females with 466 μM mifepristone rescued life span back to near virgin levels, as expected, yielding increases in mated female life span of +76% and +150%, respectively. Treatment of the mated females with 50 μM rapamycin also produced significant increases in life span of +68% and +89%, respectively. However, 100 μM rapamycin did not produce a statistically significant rescue in either experiment, suggesting it is above the optimal concentration.

**Table 1.** Statistical summary for the assay of rapamycin at 50 μM and 100 μM. Mif concentration is 466 μM. The μM concentrations of rapamycin are indicated in parentheses. Percent change in median comparisons is in the order: VF vs MF, MF vs VF in parentheses, MF + mif vs MF, MF + rap (50) vs MF, MF + rap (100) vs MF, mif + rap (50) vs MF, mif + MF + rap (50) vs MF + rap (50) in parentheses, MF + mif + rap (100) vs MF, MF + mif + rap (100) vs MF + rap (100) in parentheses. The statistical test is log-rank, and the Bonferroni-corrected *p* value for significance with six comparisons is *p* < 8.3 × 10^−3^. Significant *p* values are indicated in bold font.

Experiment	Status	Drug	N	Med	90% Mort	ΔMed (%)	*p*
1	VF	-	119	79	86	92.7	**2.13 × 10**^**−18**^
1	MF	-	122	41	72	(−48.1)	
1	MF	Mif	122	72	83	75.6	**1.22 × 10**^**−13**^
1	MF	Rap (50)	119	69	81	68.3	**1.51 × 10**^**−7**^
1	MF	Rap (100)	116	38	72	−7.31	0.426
1	MF	Mif + Rap (50)	120	39	58	−4.88 (−43.5)	5.27 × 10^−2^ (**2.27 × 10**^**−24**^)
1	MF	Mif + Rap (100)	114	37	48	−9.76 (−2.63)	**2.48 × 10**^**−5**^ (**7.05 × 10**^**−8**^)
2	VF	-	110	79	88	182	**7.82 × 10**^**−17**^
2	MF	-	115	28	77	(−64.6)	
2	MF	Mif	118	70	91	150	**7.83 × 10**^**−16**^
2	MF	Rap (50)	116	53	79	89.3	**2.0 × 10**^**−3**^
2	MF	Rap (100)	111	46	72	64.3	4.63 × 10^−2^
2	MF	Mif + Rap (50)	120	35	56	25.0 (−34.0)	0.531 (**1.06 × 10**^**−10**^)
2	MF	Mif + Rap (100)	118	39	56	39.28 (−15.2)	0.7849 (**4.41 × 10**^**−8**^)

Combining mifepristone with either 50 μM rapamycin or 100 μM rapamycin did not produce further increases in life span, indicating that the life span benefits of the drugs are not additive. Instead, combining the two drugs decreased median life span relative to mated females treated with either drug alone. We note that treating the mated females with mifepristone plus rapamycin does not consistently reduce median life span to less than that of the untreated mated females. In the first experiment (Figure 1, Table 1), treating the mated females with mifepristone plus 100 μM rapamycin reduced median life span by −9.76% relative to untreated mated females. The other comparison of mated females treated with mifepristone plus 100 μM rapamycin to untreated mated females, and the two comparisons of mated females treated with mifepristone plus 50 μM rapamycin to untreated mated females were not significant. We interpret these results to indicate that combining mifepristone and rapamycin at or near their optimal concentrations eliminates the beneficial effect of the drugs on mated female life span, but is generally not further toxic to the mated females.

To further evaluate the effects of mifepristone and rapamycin on survival, the data from the two replicate experiments were combined for a Cox proportional hazard analysis (Cox-PHA) (Table 2). The results of the Cox-PHA were consistent with the log-rank tests discussed above. The parameter estimates are for the ln(hazard rate), which indicates risk of death. A positive estimate indicates increased risk of death, whereas a negative estimate indicates reduced risk of death. Mating caused a significant increase in risk of death, whereas treatment of the mated females with mifepristone or with 50 μM rapamycin caused a significantly reduced risk of death. Treatment with 100 μM rapamycin did not produce a significant effect, consistent with the conclusion that 100 μM rapamycin is above the optimal concentration in this experiment. Combining mifepristone with either 50 μM rapamycin or 100 μM rapamycin produced a significantly increased risk of death.

**Table 2.** Cox proportional hazard analysis for assay of rapamycin at 50 μM and 100 μM. Data from Figure 1. The test is Cox regression with Efron’s approximation. Time variable = day, and number events = 1640. Mifepristone (mif) concentration is 466 μM. The μM concentrations of rapamycin (rap) are indicated in parentheses. Parameter estimates indicate ln(hazard rate).

Parameter estimates	Variable	Estimate	Standard error	95% CI
β1	Mating	1.358	0.09439	1.173 to 1.543
β2	Mif	−1.211	0.09413	−1.396 to −1.027
β3	Rap 50	−0.616	0.09293	−0.7982 to −0.4337
β4	Rap 100	−0.02447	0.09511	−0.2110 to 0.1620
β5	Mif : Rap (50)	2.408	0.1389	2.136 to 2.680
β6	Mif : Rap (100)	1.914	0.1371	1.645 to 2.183
**Hazard ratios**	**Variable**	**Estimate**	**95% CI**	
exp (β1)	Mating	3.888	3.232 to 4.680	
exp (β2)	Mif	0.2978	0.2476 to 0.3581	
exp (β3)	Rap (50)	0.5401	0.4501 to 0.6481	
exp (β4)	Rap (100)	0.9758	0.8097 to 1.176	
exp (β5)	Mif : Rap (50)	11.11	8.463 to 14.59	
exp (β6)	Mif : Rap (100)	6.778	5.182 to 8.869	
**Sig. diff. than zero?**	**Variable**	**|Z|**	**P value**	
β1	Mating	14.39	<1 × 10^−4^	
β2	Mif	12.87	<1 × 10^−4^	
β3	Rap (50)	6.629	<1 × 10^−4^	
β4	Rap (100)	0.2573	0.797 (ns)	
β5	Mif : Rap (50)	17.33	<1 × 10^−4^	
β6	Mif : Rap (100)	13.96	<1 × 10^−4^	
**Hypothesis tests**	**Statistic**	**P value**	**Reject Null Hypothesis?**	
Log-likelihood ratio (G squared)	661.6	<1 × 10^−4^	Yes	
Wald test	614.6	<1 × 10^−4^	Yes	
Score test	688.8	<1 × 10^−4^	Yes	

**Figure 2.**
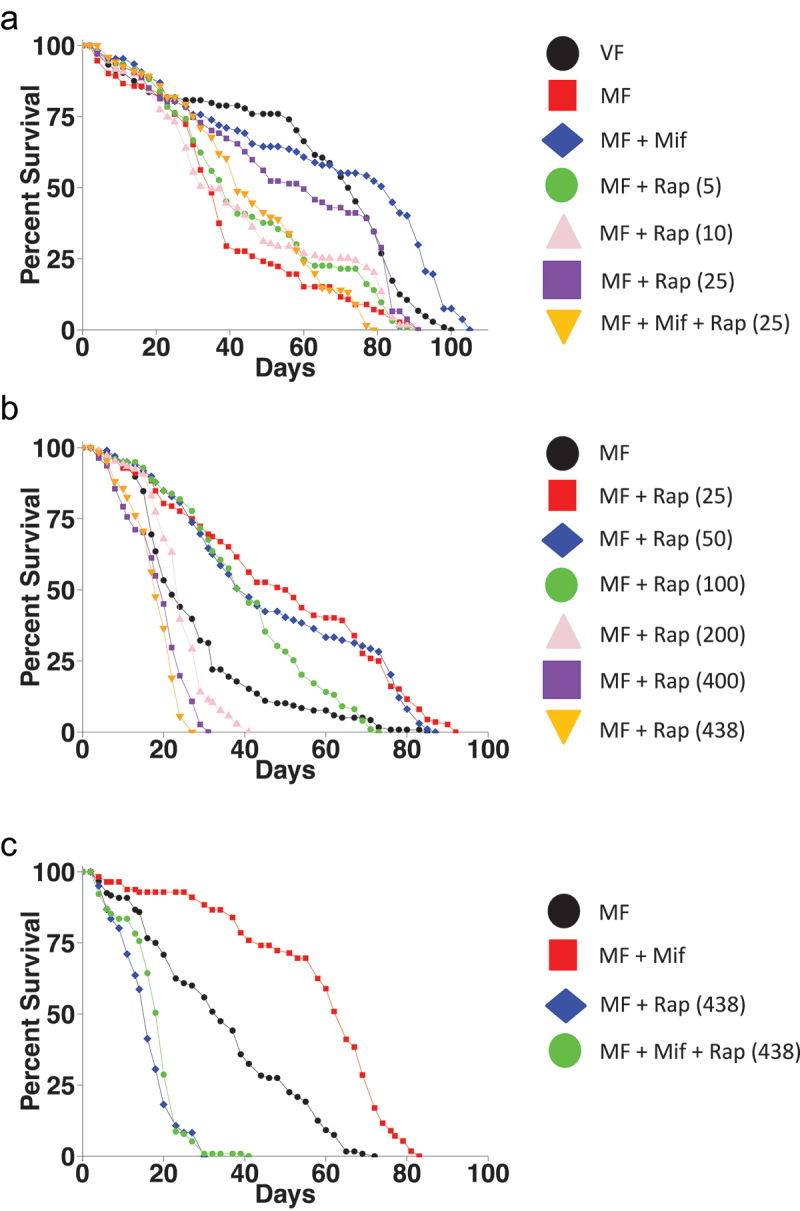
Further titration of rapamycin and non-additive benefits in combination with mifepristone. (a) titration of lower rapamycin concentrations. Life span was assayed in virgin females (VF), mated females (MF), and in mated females treated with mifepristone (mif, 466 μM), and/or rapamycin (Rap), with the μM concentrations of rapamycin indicated in parentheses. Statistical summary is presented in Table 3. (b) titration of higher rapamycin concentrations. Life span was assayed in mated females (MF), and in mated females treated with the indicated concentrations of rapamycin, with the μM concentrations of rapamycin indicated in parentheses. Statistical summary is presented in Table 4. (c) high concentration rapamycin negates the beneficial effect of mifepristone on life span. Life span was assayed in mated females (MF), and in mated females treated with mifepristone (mif, 466 μM), rapamycin (Rap, 438 μM), or mifepristone plus rapamycin, as indicated. Statistical summary is presented in Table 5.

Next, lower concentrations of rapamycin were tested (Figure 2a; Table 3). As expected, mating decreased female life span, and treatment with 466 μM mifepristone increased mated female life span by +140%. Treatment of the mated females with either 5 μM rapamycin or 10 μM rapamycin had no significant benefit, indicating these concentrations are below the optimum. By contrast, 25 μM rapamycin increased mated female life span by +71%, similar to the efficacy observed above for 50 μM rapamycin. Notably, combining 25 μM rapamycin with mifepristone did not produce further increases in life span, and instead decreased the median life span relative to either drug alone. To further test the optimal concentration of rapamycin, concentrations ranging from 25 μM to 438 μM were assayed for ability to increase mated female life span (Figure 2b; Table 4). This experiment yielded significant increases in life span for 25 μM, 50 μM and 100 μM rapamycin, whereas concentrations of 200 μM or greater were ineffective or had increasingly negative effects. Finally, 466 μM mifepristone was unable to rescue the negative effects of high concentration (438 μM) rapamycin (Figure 2c; Table 5).

**Table 3.** Statistical summary for assay of lower rapamycin concentrations. Mif concentration is 466 μM. The μM concentrations of rapamycin are indicated in parentheses. Percent change in median comparisons is in the order: VF vs MF, MF vs VF in parentheses, MF + mif vs MF, MF + rap (5) vs MF, MF + rap (10) vs MF, MF + rap (25) vs MF, MF + mif + rap (25) vs MF, MF + mif + rap (25) vs MF + rap (25) in parentheses. The statistical test is log-rank, and the Bonferroni-corrected *p* value for significance with six comparisons is *p* < 8.3 × 10^−3^. Significant *p* values are indicated in bold font.

Status	Drug	N	Med	90% Mort	ΔMed (%)	*p*
VF	-	104	73	90	108.6	**1.08 × 10**^**−10**^
MF	-	112	35	74	(−52.1)	
MF	Mif	107	84	98	140	**1.49 × 10**^**−14**^
MF	Rap (5)	93	39	81	11.4	0.1404
MF	Rap (10)	119	35	84	0.00	0.1778
MF	Rap (25)	107	60	84	71.4	**5.08 × 10**^**−6**^
MF	Mif + Rap (25)	121	42	74	20.0 (−30.0)	0.2158 (**2.35 × 10**^**−8**^)

**Table 4.** Statistical summary for titration of higher rapamycin concentrations. The μM concentrations of rapamycin indicated in parentheses. Percent change in median comparisons is in the order of each concentration of rapamycin compared to MF. The statistical test is log-rank, and the Bonferroni-corrected *p* value for significance with six comparisons is *p* < 8.3 × 10^−3^. Significant *p* values are indicated in bold font.

Status	Drug	N	Med	90% Mort	ΔMed (%)	*p*
MF	-	118	22	49		
MF	Rap (25)	112	51	83	131.8	**8.28 × 10**^**−13**^
MF	Rap (50)	99	41	80	86.3	**2.31 × 10**^**−10**^
MF	Rap (100)	99	41	64	86.4	**6.13 × 10**^**−5**^
MF	Rap (200)	106	24	33	9.09	3.49 × 10^−2^
MF	Rap (400)	111	20	29	−9.09	**2.34 × 10**^**−7**^
MF	Rap (438)	126	18	24	−18.2	**5.35 × 10**^**−10**^

**Table 5.** Statistical summary for assay of rapamycin at 438 μM. Mif concentration is 466 μM. Rap concentration is 438 μM. Percent change in median comparisons is in the order: MF + mif vs MF, MF + rap vs MF, MF + mif + rap vs MF, MF + mif + rap vs MF + rap in parentheses. The statistical test is log-rank, and the Bonferroni-corrected *p* value for significance with three comparisons is *p* < 1.67 × 10^−2^. Significant *p* values are indicated in bold font.

Status	Drug	N	Med	90% Mort	ΔMed (%)	*p*
MF	-	120	34	60		
MF	Mif	112	65	76	91.2	**2.10 × 10**^**−19**^
MF	Rap	121	16	25	−52.9	**9.29 × 10**^**−22**^
MF	Mif + Rap	115	20	23	−41.2 (25.0)	**3.73 × 10**^**−17**^ (7.38 × 10^−2^)

**Figure 3.**
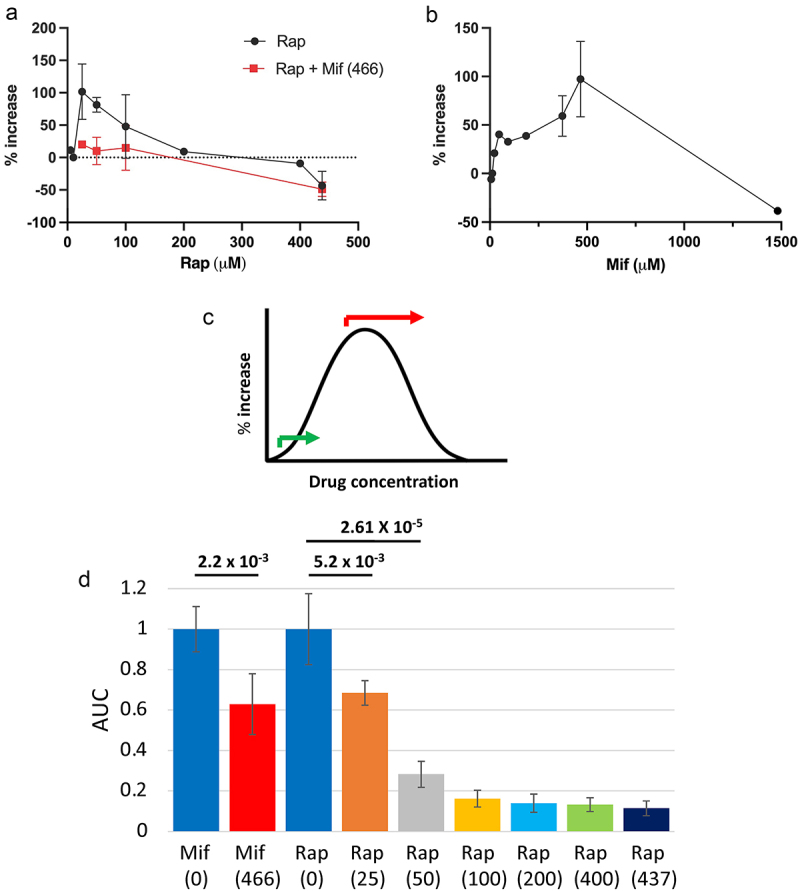
Estimation of optimal drug concentrations and inhibition of egg production. (a) estimation of optimal rapamycin concentration. The percent increase in median life span of mated females caused by rapamycin is plotted as a function of rapamycin concentration (black circles). The percent increase in median life span of mated females caused by the indicated concentration of rapamycin combined with 466 μM mifepristone is plotted as a function of rapamycin concentration (red squares). Error bars represent standard deviation. (b) estimation of optimal mifepristone concentration. The present data from Tables 1,3,4,5 is combined with a meta-analysis of previous assays of mifepristone (Supplementary Table S1). The percent increase in median life span of mated females caused by mifepristone is plotted as a function of mifepristone concentration. Error bars represent standard deviation. (c) diagram of an idealized dose–response curve for the effect of drug on mated female life span. Doubling the concentration of drug at or near the optimum is expected to reduce the response, as indicated by the red arrow. Combining two drugs at concentrations far below their optima can potentially increase the response, as indicated by green arrow. Bell curve adapted from bell curve by Lorenzo from noun project (CC by 3.0). (d) egg production. Five replicate vials of 20 mated females each were assayed every other day from day 2 of drug treatment to day 20 of drug treatment, and AUC analysis was used to estimate total egg production per vial. The concentration of drug in μM is presented in parentheses. Mifepristone assayed at 466 μM is plotted alongside rapamycin assayed at various concentrations. Drug-treated groups are compared to the corresponding no-drug control normalized to 1.0. The statistical test is unpaired, two-tailed t test, and the Bonferroni-corrected *p* value for significance with two comparisons is *p* < 2.5 × 10^−2^. Significant *p* values are indicated in bold font. Error bars represent standard deviation.

Plotting the percent increase in mated female life span caused by rapamycin treatment versus the concentration of rapamycin suggests an optimal concentration centred around 25 μM to 50 μM (Figure 3a). Both 25 μM rapamycin and 50 μM rapamycin consistently increased mated female life span across all experiments. By contrast, 100 μM rapamycin significantly increased mated female life span in one experiment (Figure 2b, Table 4), but not in two other experiments (Figure 1, Table 1), suggesting that 100 μM rapamycin is somewhat above the optimum. To estimate the optimal concentration of mifepristone, the assays of mifepristone conducted here were combined with a meta-analysis of previous mifepristone titrations [24,26,54] (Supplemental Table S1). The data were generated using the same hybrid genotype as is used here, as well as the long-lived progeny of a cross of two other laboratory strains [24] (Supplemental Table S1). Plotting the percent increase in mated female life span caused by mifepristone treatment versus the concentration of mifepristone suggests an optimal concentration of approximately 466 μM (Figure 3b). Notably, this may be an underestimate of the optimal mifepristone concentration. The data points and the shape of the curve on the ascending side of the mifepristone titration suggest that 466 μM is likely to be at or near the optimum, however, the paucity of points on the descending side of the titration curve make it possible that even greater life span increase might be observed with concentrations somewhat greater than 466 μM (Figure 3b).

Plotting the percent increase in life span resulting from combining 466 μM mifepristone with various concentrations of rapamycin illustrates that this does not yield further increases in life span. Instead, combining the two drugs significantly reduces life span relative to the effect of either drug alone, to approximately the level of untreated mated females (indicated by dotted line, Figure 3a). The lack of an additive effect of combining rapamycin and mifepristone at concentrations at or near their respective optima suggests that the two drugs act in the same pathway. The fact that combining the two drugs at or near their optimal concentrations resulted in reduced life span relative to either drug alone is consistent with this conclusion. If the two drugs act in the same pathway, then combining the two drugs at or near their optimal concentrations is equivalent to doubling the effective concentration of drug. Doubling the effective concentration of drug at or near the optimal concentration is expected to shift the dose-response into the descending region of the titration curve (indicated by red arrow, Figure 3c).

Finally, consistent with previous studies [24,29,48], both mifepristone and rapamycin significantly reduced, but did not eliminate, egg production (Figure 3d). Because 50 μM rapamycin yielded a consistent life span increase of average +81% across replicate experiments, this concentration was chosen for further experiments.

### Rapamycin reduces mating-induced midgut hypertrophy equivalent to mifepristone

**Figure 4.**
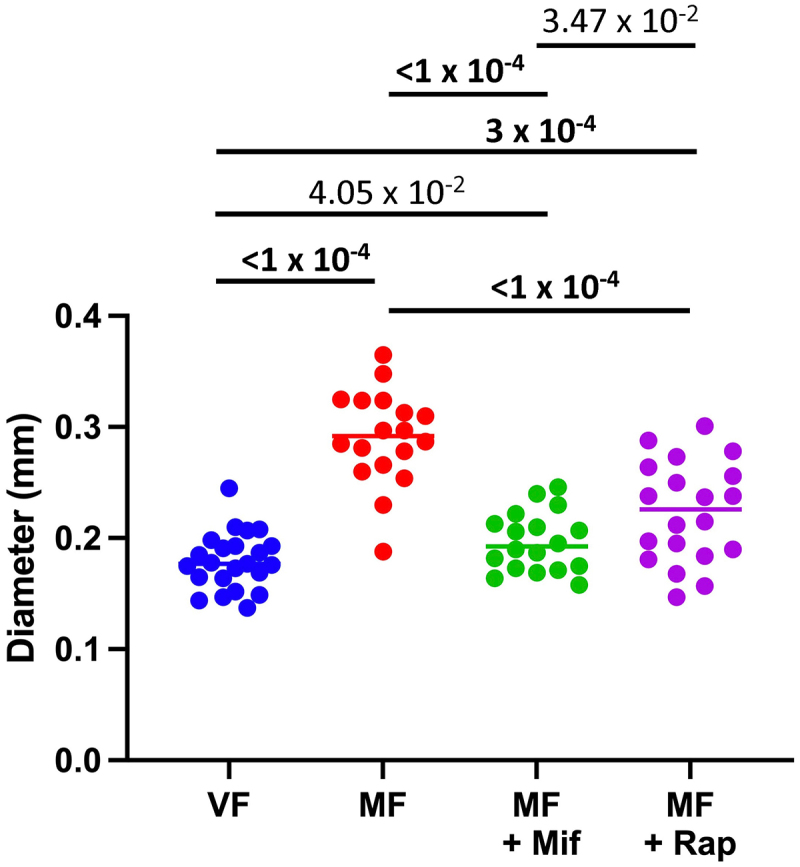
Both mifepristone and rapamycin inhibit midgut hypertrophy. Maximum midgut diameter was assayed in virgin females (VF), mated females (MF), and in mated females treated with mifepristone (mif, 466 μM) or rapamycin (rap, 50 μM), at age 14 days. The statistical test is unpaired, two-tailed t test, and the Bonferroni-corrected *p* value for significance with three comparisons is *p* < 1.67 × 10^−2^. Significant *p* values are indicated in bold font.

Previous studies show that mating causes midgut hypertrophy in the mated female, and that feeding the mated females with 466 μM mifepristone prevents this hypertrophy [28–30]. Here, 50 μM rapamycin was compared to 466 μM mifepristone for the relative ability to reduce midgut hypertrophy. Assay of maximum midgut diameter reveals a robust hypertrophy due to mating, that was blocked by feeding the mated females with 466 μM mifepristone, as expected (Figure 4). Feeding the mated females with 50 μM rapamycin also significantly reduced midgut hypertrophy, yielding a maximum midgut diameter that was not significantly different than that observed for the flies treated with mifepristone (Figure 4). These results indicate that both mifepristone and rapamycin reduce mating-induced midgut hypertrophy.

### Mifepristone activates the mito-QC mitophagy reporter equivalent to rapamycin in live flies

**Figure 5.**
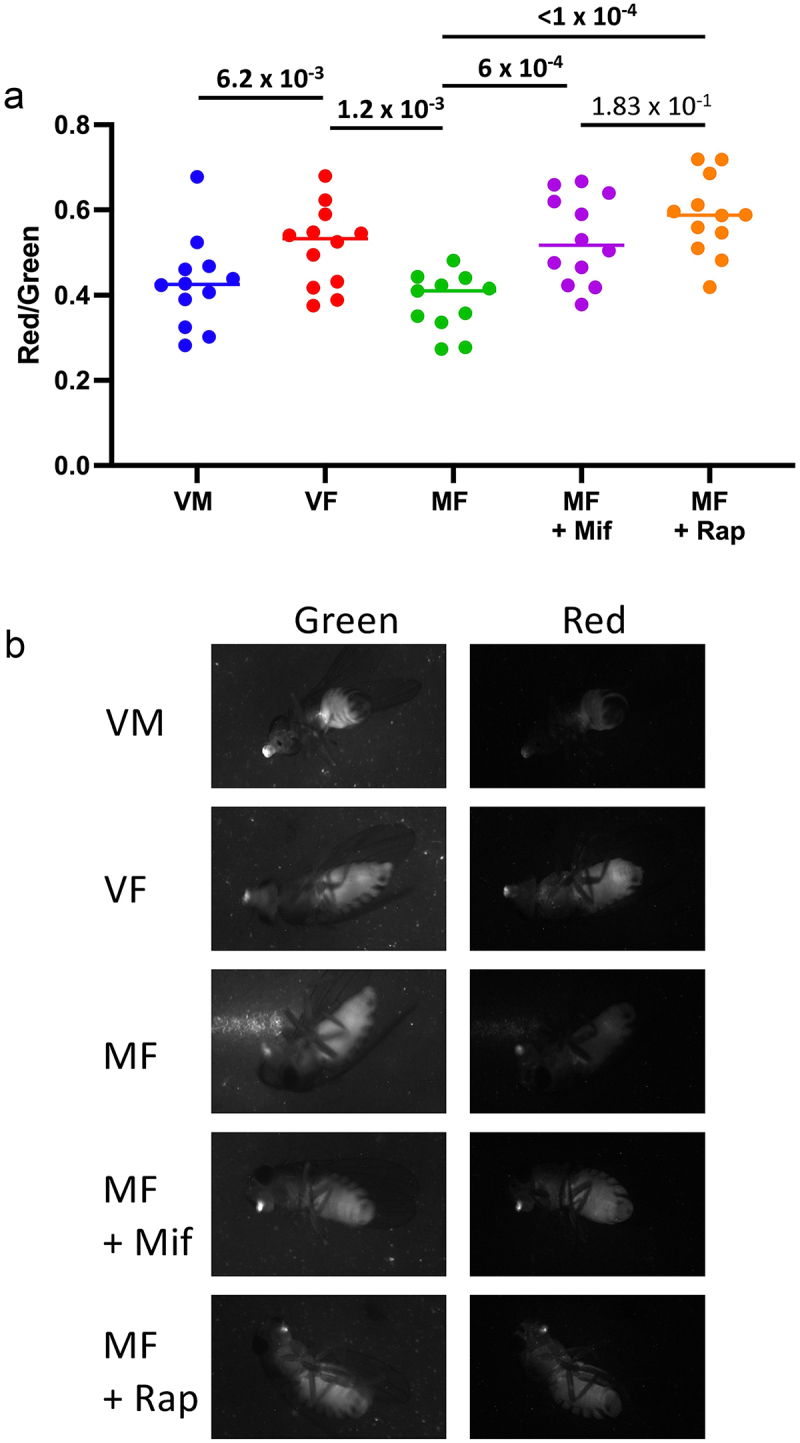
Both mifepristone and rapamycin activate the mito-QC mitophagy reporter in fat-body tissue. The fat-body specific *ppl-GAL4* driver was used to drive expression of multi-copy UAS-mito-QC transgenes. Both red and green fluorescence was assayed in virgin males (VM), virgin females (VF), mated females (MF) and in mated females treated with mifepristone (mif, 466 μM) or rapamycin (rap, 50 μM), at age 14 days. (a) the red/green ratio is plotted for each fly from each group. The statistical test is unpaired, two-tailed t test, and the Bonferroni-corrected *p* value for significance with three comparisons is *p* < 1.67 × 10^−2^. Significant *p* values are indicated in bold font. (b) green and red images are presented for a representative fly from each group, as indicated.

As discussed above, increasing evidence implicates impaired mitophagy in ageing phenotypes across species [63,64]. For this reason, it was of interest to ask if altered mitophagy might be associated with the life span effects of mating, mifepristone and rapamycin in female *Drosophila*. To begin to address this question, the mito-QC mitophagy reporter was utilized. The mito-QC mitophagy reporter is a previously described GFP–mCherry fusion protein targeted to the outer mitochondrial membrane [35,65]. Inhibition of GFP fluorescence by the acidic environment of the lysosome yields an increased red/green fluorescence ratio, indicative of increased mitophagy. To facilitate assay of the mito-QC mitophagy reporter in live adult flies, a multi-copy mito-QC strain was generated (Supplemental Figure S1). The existing transgenic insertions on the second and third chromosomes were mobilized using P element transpose to double their copy number, and these multi-insert chromosomes were then combined into the same strain. The resultant multi-copy strain *yw; UAS-mitoQC2-2; UAS-mitoQC3-2* was then crossed to the *w;ppl-GAL4* driver strain. The *ppl-GAL4* driver is reported to yield high-level expression of GAL4 in the adult fat-body, with some additional weak expression in the gut and malphigian tubules [66]. The progeny resulting from the cross contains the *ppl-GAL4* driver and four copies of the mito-QC reporter (Supplemental Figure S1). *Drosophila* males have been reported to have higher basal levels of macroautophagy in the midgut relative to females [49]. By contrast, whether males might have different basal levels of mitophagy relative to females in fat body or midgut remains unexplored. Here, virgin males, virgin females, mated females, and mated females treated for 12 days with either 466 μM mifepristone or 50 μM rapamycin were then anesthetized with CO_2_ gas, and assayed for whole-body green and red fluorescence using image capture (Figure 5). Fluorescence was detected throughout the body, including strong expression in the abdomen, consistent with fat-body and intestinal tissues (Figure 5b). In addition, strong expression was detected in the labellum. Plotting the red/green fluorescence ratio indicates greater mitophagy reporter activation in virgin females relative to virgin males, and reduced mitophagy reporter activation in mated females relative to virgin females (Figure 5a). Notably, feeding the mated females with either 466 μM mifepristone or 50 μM rapamycin produced an equivalent increase in mitophagy reporter activation, to levels equal to that of virgin females. These results indicate that both mifepristone and rapamycin block the observed mating-induced decrease in mitophagy.

### Mifepristone activates the mito-QC mitophagy reporter equivalent to rapamycin in midgut

**Figure 6.**
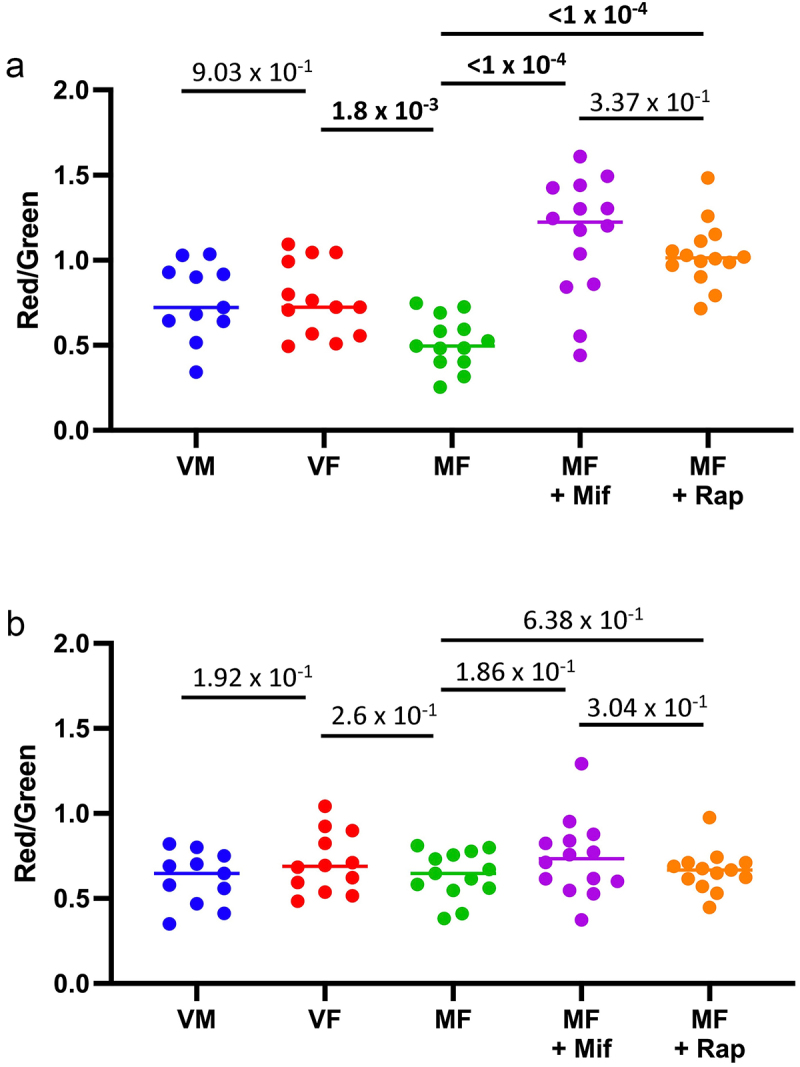
Both mifepristone and rapamycin activate the mito-QC mitophagy reporter in midgut tissue. The tissue-general *tubulin-GAL4* driver was used to drive expression of multi-copy *UAS-mito-QC* transgenes. The midgut and hindgut tissue was dissected from each fly, red and green fluorescence images were captured by fluorescence microscopy, and the red and green fluorescence was quantified in the midgut and in the hindgut using ImageJ. Fluorescence was assayed in gut tissue isolated from virgin males (VM), virgin females (VF), mated females (MF) and in mated females treated with mifepristone (mif, 466 μM) or rapamycin (rap, 50 μM), at age 14 days. (a, b) the red/green ratio is plotted for each tissue sample from each group. (a) midgut. (b) hindgut. The statistical test is unpaired, two-tailed t test, and the Bonferroni-corrected *p* value for significance with three comparisons is *p* < 1.67 × 10^−2^. Significant *p* values are indicated in bold font.

**Figure 7.**
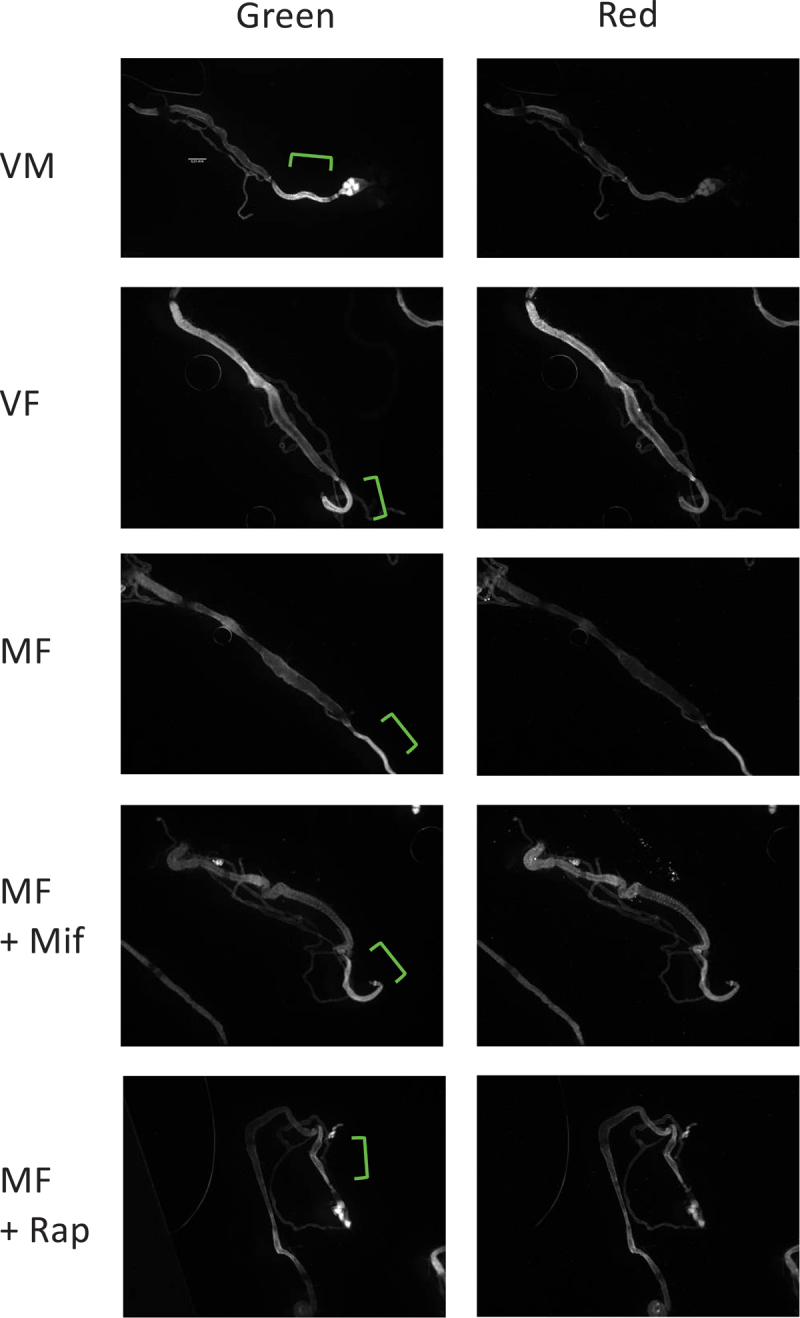
Expression of mito-QC reporter in dissected midgut tissue. Examples of green and red fluorescence images of dissected midgut tissue are presented. The examples are from the data of figure 6A. Anterior (midgut) is to the left, posterior (hindgut) is to the right. The hindgut region is marked by brackets. All images are to scale.

To assay the mito-QC reporter in dissected intestinal tissues, the multi-copy mito-QC strain was crossed to the tissue-general *yw;Tub-GAL4* driver strain to generate progeny containing the *yw;Tub-GAL4* driver and four copies of the mito-QC reporter. Virgin males, virgin females, mated females, and mated females treated for 12 days with either 466 μM mifepristone or 50 μM rapamycin were dissected in phosphate buffered saline solution, and the midgut plus hindgut tissue was mounted on slides and assayed for green and red fluorescence using image capture. The red/green fluorescence ratio for the midgut region is plotted to allow comparison between the groups (Figure 6a), and representative images of the dissected tissues are presented (Figure 7). No significant difference was detected between virgin males and virgin females. Comparing virgin females to mated females revealed a reduction in mitophagy reporter activation due to mating. Notably, feeding the mated females with either 466 μM mifepristone or 50 μM rapamycin produced a significant increase in mitophagy reporter activation, to levels even greater than that observed in virgin females. There was no significant difference in the level of mitophagy reporter activation by mifepristone versus rapamycin, indicating that these drugs produce equivalent activation of mitophagy in the midgut.

The mito-QC reporter activation was also assayed for the hindgut region (Figure 6b; Figure 7). Whereas considerable variation in hindgut fluorescence was detected between samples, there was no significant difference in the red/green ratio between the groups, indicating that neither mating nor drug treatment significantly alters mitophagy in the hindgut.

## Discussion

Here, the drugs mifepristone and rapamycin were compared for their relative ability to increase the life span of mated female *Drosophila melanogaster*. Titration of rapamycin indicated an optimal concentration of approximately 50 μM, which increased median life span here by average +81%. Meta-analysis of previous mifepristone titrations indicated an optimal concentration of approximately 466 μM, which increased median life span here by average +114%. Combining mifepristone with various concentrations of rapamycin did not produce further increases in life span, and instead reduced life span relative to either drug alone, to levels similar to that of untreated mated females (Figure 3a). The lack of an additive effect when the drugs are combined at or near their optimal concentrations indicates that the drugs act in the same pathway. Consistent with this conclusion, the combination of the two drugs at or near their optimal concentration resulted in reduced life span relative to either drug alone. If the two drugs act in the same pathway, then combining the two drugs at or near their optimal concentrations is equivalent to doubling the effective concentration of drug. Doubling the effective concentration of drug at or near the optimal concentration is expected to shift the dose-response into the descending region of the titration curve (indicated by red arrow, Figure 3c). We note that combining mifepristone with rapamycin did not consistently reduce median life span to less than that of untreated mated females. In one assay of mifepristone combined with 100 μM rapamycin, the median life span of the treated mated females was reduced by −9.76% relative to untreated mated females (Figure 1, Table 1). By contrast, the replicate assay of mifepristone combined with 100 μM rapamycin, and the two assays of mifepristone with 50 μM rapamycin did not significantly reduce life span relative to untreated mated females (Figure 1, Table 1). Similarly, combining mifepristone with 25 μM rapamycin did not significantly reduce life span to less than untreated mated females (Figure 2, Table 3). These results are summarized in a plot of the percent increase in mated female life span versus rapamycin drug concentration, where the dotted line indicates the baseline life span of untreated mated females (Figure 3a). The observation that combining the two drugs reduces mated female life span to approximately the same level as the untreated mated females suggests that the benefits for mated female life span are lost, but that the drug combination is not further toxic to the mated females. Finally, it is possible that combining mifepristone with rapamycin at concentrations far below their optima might increase life span, by shifting the response further up the ascending side of the dose–response curve (indicated with green arrow, Figure 3c). However, such a result would not distinguish between drugs acting in the same pathway as opposed to drugs acting in different pathways.

Interestingly, the magnitude of life span increase observed here for rapamycin is greater than previous reports, where rapamycin typically increased mated female life span by less than 30% [48,50,67]. This difference is likely due to the use of a hybrid genetic background that is particularly sensitive to the negative life span effect of mating, allowing for a greater magnitude life span increase when the negative effect of mating is rescued by the drug. It is also notable that the optimal concentration for rapamycin is about one-tenth the estimated optimal concentration for mifepristone. This might indicate that mifepristone has a lower affinity for a common binding site on a target protein, or alternatively, that rapamycin and mifepristone might target different proteins that function in the same pathway, and/or there may be differences in drug uptake and bioavailability. Consistent with the conclusion that mifepristone and rapamycin target the same pathway, the two drugs have additional similarities in their effects. Rapamycin was found to be equally efficacious as mifepristone in blocking the midgut hypertrophy caused by mating. Moreover, mifepristone was equally efficacious as rapamycin in activating the mito-QC mitophagy reporter in fat-body and midgut.

During mating in *Drosophila*, a seminal hormone called Sex Peptide is transferred into the female reproductive tract from the male [68]. The Sex Peptide acts in the female to cause increased levels of the hormones ecdysone and juvenile hormone (JH). These hormones in turn induce midgut hypertrophy, inflammation markers, increased AA and lipid metabolism, and increased egg production [23,25,26,69–74]. These changes are largely or completely blocked by feeding the mated females mifepristone, leading to dramatic increases in mated female life span [24,26,27] (this study). By comparing wild-type males to males with null mutation of the Sex Peptide gene, and using the same long-lived hybrid female genotype as is used here, it was shown that the majority of the life span increase due to mifepristone treatment in mated females is due to the rescue of the negative life span effect of male Sex Peptide [26]. Moreover, feeding virgin female flies with a potent analog of JH called methoprene [25,75], or feeding virgin female flies with a potent mimic of ecdysone called RH5849 [69,76], recapitulated the dramatic decrease in life span caused by mating, and these decreases were significantly rescued by feeding the flies with mifepristone, without reducing food intake [27,30]. Taken together, these studies indicate that mifepristone acts to block the effects of male Sex Peptide on female life span and other phenotypes. As discussed above, the non-additive effects of mifepristone and rapamycin on life span, and their similar effects on midgut hypertrophy and the mitophagy reporter suggest that mifepristone and rapamycin act to inhibit the same pathway. This hypothesis predicts that, like mifepristone, rapamycin will increase life span to a greater extent in mated females than in virgin females, and that a large part of the rapamycin life span increase in mated females will be dependent upon the presence of a functional Sex Peptide gene in the males used for mating. In the future, it may be of interest to test these predicted properties of rapamycin.

The conditional gene expression system called Gene-Switch [77–80], is activated by feeding flies mifepristone, and has been used to knock-down gene expression in the midgut to ask which factors might be required for life span extension by rapamycin [67,81]. Factors identified in this way include increased histone H3/H4 expression, the autophagy regulators Atg1 and Atg5, and the transcription factor encoded by the gene *fork head*. The observation that combining mifepristone with rapamycin can reduce or eliminate the life span benefits of rapamycin (Figure 3a) suggests that caution should be taken when interpreting experiments that use the Gene-Switch system to study the effects of rapamycin. In the future, it may of interest to determine if mifepristone and rapamycin require the same factors in the midgut to increase life span. In mammals, mifepristone binds and activates the transcription factor PPARγ [4,5]. *Drosophila* contains an ortholog of PPARγ called Eip75B [82]. Recently, a conditional gene expression system based on FLP recombinase was used to inhibit Eip75B activity in a tissue-general pattern, and this was found to eliminate the life span effects of mating and mifepristone [83]. In the future, it may be of interest to ask what are the critical tissues where Eip75B is needed to mediate life span extension by mifepristone, and to ask whether Eip75B might also be required for life span extension by rapamycin.

## Supplementary Material

Landis Fly Supplementary Materials RV4.docx

## Data Availability

All data is available upon reasonable request to the corresponding author.
