# Supplementary material for: Mifepristone and rapamycin have non-additive benefits for life span in mated female Drosophila
Source: Fly (Austin). 2024 Oct 23;18(1):2419151. doi: 10.1080/19336934.2024.2419151 (PMC11514543; doi:10.1080/19336934.2024.2419151)
Supplement: Landis Fly Supplementary Materials RV4.docx [file KFLY_A_2419151_SM0828.docx]

**Mifepristone and rapamycin have non-additive benefits for life span in mated female**

***Drosophila***

Gary N. Landis, Britta Baybutt, Shoham Das, Yijie Fan, Kate Olsen, Karissa Yan, John Tower

**Supplementary materials**

**Supplemental Table S1**. Data summary for meta-analysis of mifepristone titration. The percent increase in median is relative to control mated females (not treated with drug) from each experiment. The data are plotted in Figure 3B.

| **Source** | **Genotype** | **Conc. μg/ml** | **Conc. μM** | **% inc. median** |
| --- | --- | --- | --- | --- |
| This study | *w[1118] X yw;Elav-GS* | 200 | 466 | 91.2 |
| This study | *w[1118] X yw;Elav-GS* | 200 | 466 | 75.6 |
| This study | *w[1118] X yw;Elav-GS* | 200 | 466 | 150 |
| This study | *w[1118] X yw;Elav-GS* | 200 | 466 | 140 |
| (1) | *w[1118] X yw;Elav-GS* | 200 | 466 | 56.25 |
| (1) | *w[1118] X yw;Elav-GS* | 200 | 466 | 70.4 |
| (2) | *w[1118] X yw;Elav-GS* | 160 | 373 | 94.1 |
| (2) | *w[1118] X yw;Elav-GS* | 160 | 373 | 71.4 |
| (2) | *w[1118] X yw;Elav-GS* | 160 | 373 | 68.6 |
| (2) | *w[1118] X yw;Elav-GS* | 160 | 373 | 39 |
| (3) | *w[1118] X yw;Elav-GS* | 160 | 373 | 64.3 |
| (3) | *p53B[6] X w;rtTA(3)E2* | 160 | 373 | 64.4 |
| (3) | *p53B[6] X w;rtTA(3)E2* | 160 | 373 | 30.1 |
| (3) | *p53B[6] X w;rtTA(3)E2* | 640 | 1481 | -38.4 |
| (3) | *p53B[6] X w;rtTA(3)E2* | 2.5 | 6 | -5.9 |
| (3) | *p53B[6] X w;rtTA(3)E2* | 5.0 | 12 | 0 |
| (3) | *p53B[6] X w;rtTA(3)E2* | 10 | 23 | 20.9 |
| (3) | *p53B[6] X w;rtTA(3)E2* | 20 | 47 | 40.3 |
| (3) | *p53B[6] X w;rtTA(3)E2* | 40 | 93 | 32.8 |
| (3) | *p53B[6] X w;rtTA(3)E2* | 80 | 186 | 38.8 |
| (3) | *p53B[6] X w;rtTA(3)E2* | 160 | 373 | 41.8 |

1. Landis GN, Riggan L, Bell HS, Vu W, Wang T, Wang I, et al. Mifepristone Increases Life Span in Female *Drosophila* Without Detectable Antibacterial Activity. Front Aging. 2022;3:924957.
2. Tower J, Landis GN, Shen J, Choi R, Fan Y, Lee D, et al. Mifepristone/RU486 acts in *Drosophila melanogaster* females to counteract the life span-shortening and pro-inflammatory effects of male Sex Peptide. Biogerontology. 2017;18(3):413-27.
3. Landis GN, Salomon MP, Keroles D, Brookes N, Sekimura T, Tower J. The progesterone antagonist mifepristone/RU486 blocks the negative effect on life span caused by mating in female *Drosophila*. Aging (Albany NY). 2015;7(1):53-69.

A

*UAS-mito-QC*

mini-*white*+

5’P

B

*UAS-mito-QC*

mini-*white*+

*UAS-mito-QC*

mini-*white*+

*mito-QC-2*

3’P

transposase

*mito-QC2-2*

C

*w ppl-GAL4*

;

x *yw*

*mito-QC2-2*

; ;

*mito-QC3-2*

*w ppl-GAL4*

*7 mito-QC2-2 mito-QC3-2*

*yw* ; *mito-QC2-2*; *mito-QC3-2*

*w ppl-GAL4 +*

*w* ; *mito-QC2-2*; *mito-QC3-2*

*7 ppl-GAL4 +*

**Supplemental Figure S1. Generation and crossing scheme for mito-QC reporter flies.** The mito-QC-2 and mito-QC-3 constructs are inserted in the attP16 and attP2 sites, respectively.

Because these recombination sites are flanked by P element inverted repeats, it allows for mobilization of the entire locus by P element transposase. **(A)** Partial diagram of the mito-QC-2 transgenic insertion, indicating the 5’ and 3’ P element inverted repeats, the min-*white*+ marker gene sequences, and the *UAS-mito-QC* gene sequences. **(B)** Crossing to a strain expressing P element transposase causes transposition of the construct, typically into the 5’ or 3’ P element end sequences, resulting in a duplication. Increased copy number was scored by increased expression of the mini-*white*+ marker gene, resulting in darker orange-colored eyes. Increased copy number of the *UAS-mito-QC* construct was confirmed by crossing to a *Tubulin-GAL4* strain and analysis of the progeny by fluorescence microscopy. This confirmed doubled intensity of both green and red fluorescence, and the strain was named *yw; mito-QC2-2*. The same scheme was used to double the copy number of the mito-QC-3 insertion to yield a strain name *yw; mito- QC3-2.* Each of the new strains were crossed to a double-balancer strain, and the appropriate progeny were crossed *inter se* to combine the two chromosomes into the same strain, named *yw; mito-QC2-2; mito-QC3-2*. **(C)** Crossing scheme to generate flies for analysis. Virgin females of the homozygous *w; ppl-GAL4* strain were crossed to males of the homozygous *yw; mito-QC2-2; mito-QC3-2* strain, to generate progeny containing one copy of *ppl-GAL4* construct, and total 4 copies of the *UAS-mito-QC* construct, as indicated.
